# Artificial Intelligence in Patch Testing: Comprehensive Review of Current Applications and Future Prospects in Dermatology

**DOI:** 10.2196/67154

**Published:** 2025-06-02

**Authors:** Hilary S Tang, Joseph Ebriani, Matthew J Yan, Shannon Wongvibulsin, Mehdi Farshchian

**Affiliations:** 1 Division of Dermatology, David Geffen School of Medicine, University of California, Los Angeles, Los Angeles, CA, United States

**Keywords:** machine learning, ML, artificial intelligence, AI, algorithm, model, analytics, patch testing, allergic contact dermatitis, dermatitis, dermatology, dermatologist, skin, comprehensive review, comprehensive reviews, review

## Abstract

**Background:**

The integration of artificial intelligence (AI) into patch testing for allergic contact dermatitis (ACD) holds the potential to standardize diagnoses, reduce interobserver variability, and improve overall diagnostic accuracy. However, the challenges and limitations hindering clinical implementation have not been thoroughly explored.

**Objective:**

This narrative review aims to examine the current applications of AI in patch testing, identify challenges, and propose future directions for their use in dermatology.

**Methods:**

PubMed was searched in August 2024 to identify studies involving human participants undergoing patch testing with AI used in the study. Exclusion criteria were non-English and nonoriginal research. Data were synthesized to assess study design, performance, and potential for clinical application.

**Results:**

Out of 94 reviewed articles, 10 met the inclusion criteria. Most studies employed convolutional neural networks (CNN) for image analysis, with accuracy rates ranging from 90.1% to 99.5%. Other AI models, such as gradient boosting and random forest, were used for risk prediction and biomarker discovery. Key limitations included limited sample sizes, variability in image capture protocols, and lack of standardized reporting on skin types.

**Conclusions:**

AI has significant potential to enhance diagnostic accuracy, standardize patch test interpretation, and expand access to patch testing. However, standardized imaging protocols, larger and more diverse datasets, and improved regulatory frameworks are necessary to realize the full potential of AI in patch testing.

## Introduction

Allergic contact dermatitis (ACD) is a common inflammatory skin condition affecting approximately 20% of the population, with significant impacts on patients’ quality of life and productivity [1,2]. Traditional patch testing methods, while effective for diagnosing ACD, can be time-consuming and subject to interobserver variability [3,4]. As technology continues to advance, the integration of artificial intelligence (AI) offers the possibility of standardizing interpretations, reducing human error, and potentially improving the overall diagnostic process in patch testing [5].

AI, broadly defined as the ability of computer systems to mimic human cognitive functions, encompasses various computational subfields, including machine learning (ML). Furthermore, deep learning (DL), a subset of ML, uses algorithms modeled after human neurons to detect complex patterns and relationships in data [6]. These AI technologies have shown promising applications in dermatology, ranging from identifying skin malignancies to classifying inflammatory skin conditions and analyzing clinical notes. The visual nature of dermatology, combined with the increasing volume of clinical photographs, dermoscopy images, abundance of psychometric data from wearable devices, and electronic health records, makes it particularly well-suited for AI-augmented patient care [6-8].

The use of AI in patch testing is particularly intriguing due to the complex nature of interpreting patch test results. Several factors, such as weak positive reactions, irritant reactions, and the timing of readings, can all influence the accuracy of diagnoses, leading to interobserver variability and diagnostic inconsistencies [9-12]. Furthermore, the process is time-intensive, requiring multiple clinic visits for patients, and resource-heavy for clinics, requiring 1 visit of application of allergens, an initial removal and preliminary evaluation visit around 48 hours, and a final follow-up evaluation several days later [13,14]. AI offers the potential to automate and standardize patch test result interpretations, reducing diagnostic variability and enabling broader access to this crucial diagnostic tool. AI can also analyze large datasets to uncover patterns and trends that may not be immediately evident to clinicians, ultimately enhancing the diagnostic process for ACD while mitigating bias and promoting equitable care across diverse patient populations [15,16].

This narrative review aims to explore the current landscape of AI applications in patch testing for ACD. We will examine the types of algorithms that are currently being researched, their performance, the challenges faced, and potential future directions for this rapidly evolving field. By synthesizing the available literature, we hope to provide a comprehensive overview of the state of AI in patch testing and how AI can be leveraged to improve patch testing practices and diagnostic accuracy of ACD in the future.

## Methods

### Search Strategy

A comprehensive literature search was performed in August 2024 using the PubMed database. The search was conducted without date restrictions to capture the full scope of research in this emerging field. This broad approach ensured that all relevant studies, regardless of publication date, were included, providing a more thorough evaluation of AI applications in patch testing and the observance of any trends over time. Literature searches were conducted using combinations of keywords, such as “artificial intelligence,” “machine learning,” “patch testing,” and “contact dermatitis” or “skin” (see Multimedia Appendix 1 for the full search term list). These terms were chosen to ensure a wide net was cast, incorporating both general AI terms and specific patch testing and dermatology-related concepts.

### Inclusion and Exclusion Criteria

Criteria for inclusion and exclusion were defined prior to screening to reduce potential biases. Studies were included if they met the following criteria: the population consisted of human patient populations undergoing patch testing; the study design involved AI (which includes ML and DL); and outcomes reported on the performance of these algorithms. All publication types, including journal articles, conference abstracts, and preprints, were considered. Studies were excluded if they were not written in English or if they were not original research, such as review papers or perspectives.

### Study Selection Process and Data Extraction

The PRISMA (Preferred Reporting Items for Systematic Reviews and Meta-Analyses) approach was selected to ensure transparency and replicability in the selection process, providing a clear pathway from initial search to final inclusion [17,18]. Each article was independently reviewed by 2 authors. In cases of disagreement, a third author resolved the discrepency. From the included studies, the following data elements were then extracted: study design; sample size; skin types included; length of study for each participant; location of study; materials used (such as types of allergen panels and imaging equipment); type of AI algorithm and its performance in the study; limitations and challenges of the study; and future directions. To ensure that AI models were properly evaluated, each study relied on a clearly defined ground truth as the reference standard for their data. This ground truth was established by dermatologists’ manual interpretation of patch test reactions, typically following standardized grading criteria such as the International Contact Dermatitis Research Group (ICDRG) scale, with some studies following European Society of Contact Dermatitis guidelines or similar clinical severity scales [19]. The findings were then synthesized to highlight trends, gaps, and potential areas for future research in the application of AI in patch testing. This synthesis serves as a foundation for guiding future research efforts, with the goal of synthesizing both technical and clinical factors of the clinical patch testing procedure, analysis, data capturing, image capturing and storage, AI algorithms, and diagnostic accuracy comprehensively, contributing to the current gaps in the current practice of AI integration within dermatological patch testing diagnostics.

## Results

### Included Studies

**Figure 1.**
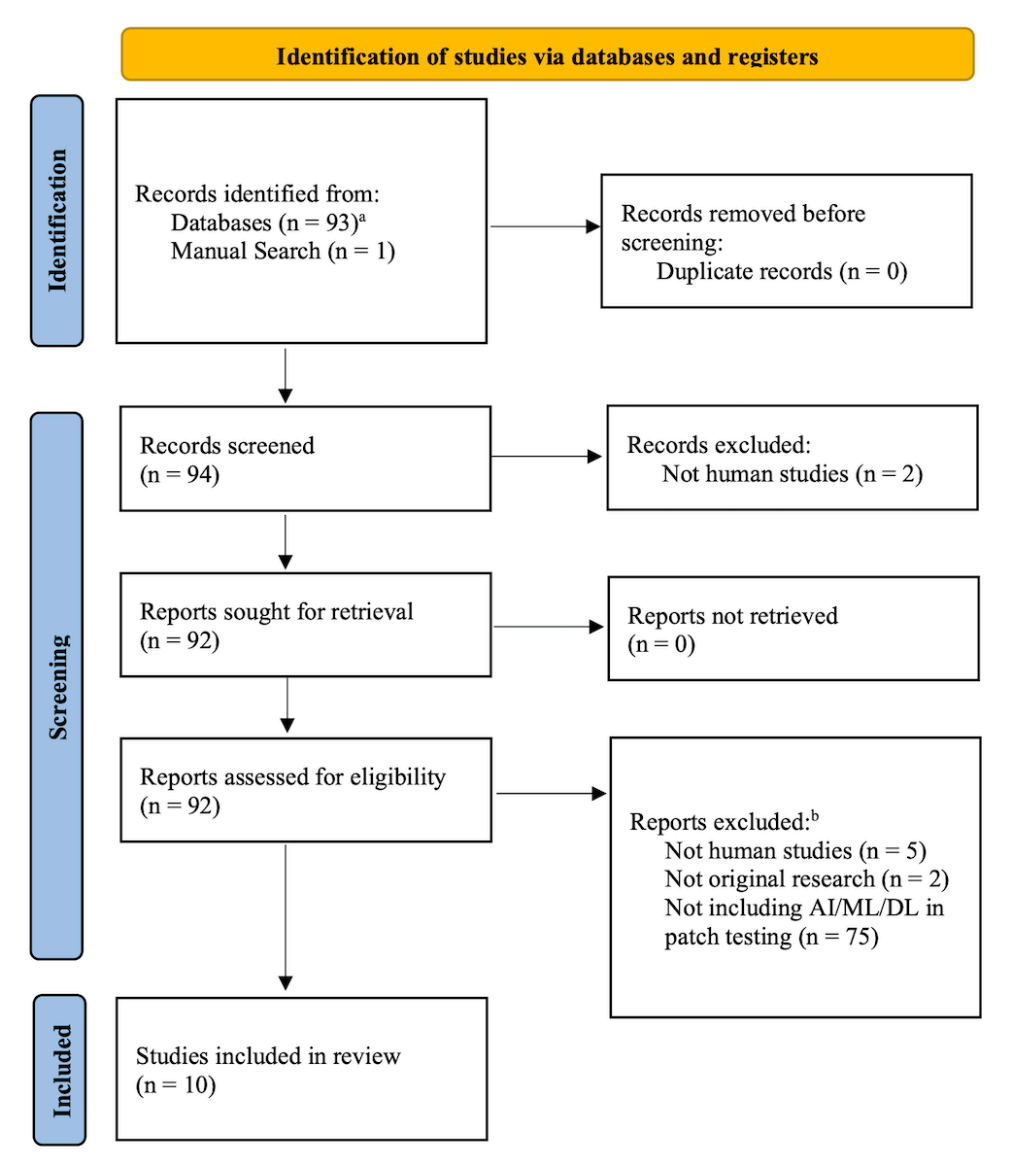
PRISMA (Preferred Reporting Items for Systematic Reviews and Meta-Analyses) 2020 flow diagram for the identification of studies [17]. ^a^Databases used in this narrative review: PubMed ^b^Reports excluded: Does not meet inclusion criteria: (1) *Population*: All patient populations (humans) undergoing patch testing; (2) *Interventions*: Study designs of artificial intelligence (AI) or machine learning (ML) or deep learning (DL) algorithms in patch testing; (3) *Outcomes*: Non-English and nonoriginal research (eg, review papers, perspectives) were excluded for the purposes of this narrative review.

A total of 94 records were ultimately screened and evaluated for eligibility, as shown in the PRISMA flow diagram (Figure 1) [17]. Of the 94 articles, our literature review identified 10 relevant studies that employed various AI techniques in the context of patch testing and skin sensitization prediction, as shown in Table 1. These studies encompassed a wide range of approaches, from image analysis of patch test results to molecular profiling and risk prediction models.

**Table 1.** Summary of articles included in this comprehensive review of artificial intelligence in patch testing.

Author, Year	Study objective	Type of AI used	Location	Fitzpatrick skin types (FST) included; Demographics, n (%)	Age in years, median (range)	Materials used	Total data sample size	Test, validation, evaluation data sample size	Accuracy, performance
Kyritsi et al, 2024 [20]	To investigate the contact allergy patterns	Multiple correspondence analysis (MCA), CATPCA (categorical principal components analysis)	Greece	Not reported	4 (18‐86)	4 allergens	800 patients; clinical, demographic, occupational data	Not reported	Not reported
Ravishankar et al, 2024 [21]	To evaluate the use of convolutional neural networks to determine presence of patch test reactions	Convolutional neural network (CNN)	United States	FST I-II: 110 (88%), III-V: 15 (12%); Caucasian: 100 (80%), Black: 4 (3.2%), Asian: 6 (4.8%), Unknown: 15 (12%)	46.8 (34.6‐60.9)	Not reported	125 patients; 13,622 images	Test set: 2725 images	Area under the curve (AUC): 0.940, accuracy: 90.1%, sensitivity: 86.0%, Specificity: 90.2%
Hall et al, 2024 [22]	To develop a deep learning algorithm for the analysis of patch testing	CNN	United States	White (typically FST I-III): 165 (82.1%), Black or African American: 20 (10.0%), Asian: 5 (2.5%), Other or unknown: 11 (5.5%)	58 (18‐103)	80 allergens (Mayo Clinic standard series) were used for all patients; specific series/panels varied by patient.	201 patients; 2810 image tiles	Evaluation set: 37 patients; 507 images	AUC: 0.885, accuracy: 90.9%, sensitivity: 70.1%, specificity: 91.7%, F1 score: 37.1
Kyritsi et al, 2023 [23]	To investigate the patterns of contact sensitization	MCA	Greece	Caucasian: 240 (100%)	39 (19‐82)	3 allergens	240 patients; clinical, demographic, occupational data	Not reported	Not reported
Vezakis et al, 2023 [24]	To investigate the feasibility of using a deep learning classifier for automating the identification of allergens causing ACD	CNN	Greece	Not reported	Not reported	30 allergens	200 patients; 1190 images	Validation set: 357 images	Preprocessing scheme comparison: F1 score: 0.83, accuracy: 90%, specificity: 95%, recall: 79%, precision: 87%
Lefevre et al, 2021 [25]	To characterize the molecular signatures of chemical-induced skin inflammation through comprehensive transcriptomic analysis	Boruta, random forest (RF)	France, Belgium	Not reported	61 (29‐88)	6 allergens, 3 irritants	47 patients; 47 patch test biopsies	Not reported	RF: accuracy: 90%‐100%
Chan et al, 2021 [26]	To develop a machine learning approach for accurate classification of patch-test photographs	CNN	United States	FST I: 2 (2.6%), II: 28 (36.4%), III: 29 (37.7%), IV: 14 (18.2%), V: 4 (5.2%)	Not reported	80 allergens (American Contact Dermatitis Society (ACDS) Core Screening Allergen Series)	77 patients; 3695 images	CNN training set: 1118 images; Validation set: 373 images; Test set: 2204 images	AUC: 0.915, accuracy: 99.5%, F1 score: 0.89
Cunningham et al, 2021 [27]	To compare the predictive accuracy of logistic regression with more sophisticated machine learning approaches such as gradient boosting in predicting patch testing results	Gradient boosting, RF, AdaBoost, logistic regression (LR)	United Kingdom	Not reported	Mean 40.2	36 allergens	42,434 patients; clinical, demographic data	Test set: 10,609 patients	Gradient boosting: AUC mean: 0.69 (SD 0.06). RF: AUC mean: 0.60 (SD 0.052). AdaBoost: AUC mean: 0.58 (SD 0.048). LR: AUC mean: 0.65 (SD 0.068).
Fortino et al, 2020 [28]	To identify and validate biomarkers to distinguish allergic and irritant contact dermatitis in human skin	GARBO^a^	Finland	Not reported	Not reported	4 allergens	85 patients; 89 patch test biopsies	Validation set: 31 patch test biopsies	Accuracy: 86%‐94%, F1 score: 94% for allergic contact dermatitis, 92% for irritant contact dermatitis
Adler et al, 2017 [29]	To identify if certain pairs of positive reactions to allergens may be associated with polysensitization	RF, LR	Germany, Switzerland, Austria	Not reported	50.7	24 allergens	105,325 patients; clinical, demographic data	Tuning set^b^: 35,294; Validation set: 70,031	LR: AUC: >0.90

a Genetic AlgoRithm for biomarker selection in high-dimensional Omics with RF-based classifier.

b Tuning set refers to a subset of data used to fine-tune the parameters of a machine learning model. In this study, the tuning set was used to optimize the hyperparameters of RF and LR models before final evaluation on the validation dataset.

### Characteristics of Included Studies

Geographically, the studies were conducted across various countries and continents, with the United States (3 studies) and Greece (3 studies) being the most represented. The remaining studies were distributed across other European countries, including the United Kingdom, Finland, and a multi-country study spanning Germany, Switzerland, and Austria. Sample sizes also varied considerably between the studies, ranging from 47 patients in the molecular signature study by Lefevre et al [25] to 105,325 patients in the large-scale analysis by Adler et al [29]. In total, 9 studies had dermatologists as authors, with some contributions including patient recruitment, clinical assessment, or patch test evaluation [20-28]. The materials used for patch testing varied, with many using standard European baseline series allergens. However, some studies, such as Lefevre et al [25] and Fortino et al [28], used specific sets of allergens and irritants for their molecular profiling approaches. Most studies classified reactions on a scale ranging from negative or irritant to +++ for strongly positive reactions, though the specific scoring systems and timepoints for evaluation varied between studies.

Of the 10 studies reviewed, 4 analyzed images, 4 analyzed clinical and demographic data, and 2 analyzed biological mechanisms of biopsies for patch testing. In total, 4 studies analyzed photographic images of patch test sites, which were captured using a range of imaging modalities [21,22,24,26]. Of these 4 studies, 3 used digital camera or smartphone camera images, while Vezakis et al [24] used an advanced multi-modal imaging device, the Antera 3D® camera, which captured 6 image modalities—color, redness, texture, fine lines, and volumes (see Multimedia Appendix 2 for expanded information on the 4 image datasets). The detailed information on skin topography and chromophore concentration, captured by the Antera 3D® camera independent of lighting, provides a standardization that improves accuracy and the need for additional standard dermoscopic image preprocessing techniques [24]. In total, 4 studies analyzed clinical and patient demographic data as predictive features for ML models, which included anatomical sites, age, gender, and sex [20,23,27,29]. Additionally, 3 studies included additional clinical parameters, such as occupation and atopic history [20,23,27]. Other clinical data included the patch test ICDRG evaluations, MOAHLFA (Male-Occupational-Atopic-Hand-Leg-Face-Age) Index, and skin characteristics [20,23]. Lastly, 2 studies analyzed genomic and molecular profiling of patch test biopsies [25,28]. Seven studies reported age groups in their studies with median or mean patient age ranges from 39 to 61 years [20-23,25,27,29]. Regarding skin types, only 3 out of the 10 studies reported on the distribution of skin tones in their datasets [21,22,26]. Chan et al [26] included Fitzpatrick skin types (FST) I-V, with the majority falling into FST II-III. Hall et al [22] reported that 82% of their patient population was White, with lighter skin tones typically ranging from FST I-III. Ravishankar et al [21] showed a significant imbalance, with 88% of images representing lighter skin tones from FST I-II.

### AI and ML Techniques Used

Overall, convolutional neural networks (CNN) were the most commonly used algorithms for image analysis of patch test reactions, employed in 4 of the 10 studies [21,22,24,26]. These CNN-based models demonstrated high accuracy in identifying and classifying patch test reactions. Hall et al [22] reported an accuracy of 90.9% with an area under the curve (AUC) of 0.885, while Chan et al [26] achieved an even higher accuracy of 99.5% with an AUC of 0.915. Similarly, Ravishankar et al [21] and Vezakis et al [24] reported accuracies of 90.1% and 90%, respectively, further supporting the potential of CNN use in patch test interpretation. Other approaches, such as random forest (RF), gradient boosting (GB), and logistic regression (LR), were employed in studies focusing on risk prediction and biomarker discovery [25,27-29]. Notably, Cunningham et al [27] compared multiple algorithms and found that GB outperformed other predictive methods, including LR, RF, and AdaBoost, with AUCs of 0.69, 0.65, 0.60, and 0.58, respectively, for predicting cutaneous allergy risk. In total, 2 studies used multiple correspondence analysis to investigate patterns and relationships in patch test data, particularly in the context of occupational dermatitis and population-specific sensitization profiles [20,23]. While these studies did not provide specific accuracy metrics, they demonstrated the utility of AI techniques in uncovering complex associations within patch test data.

## Discussion

### Principal Findings

This review of 10 studies exploring the application of AI techniques in patch testing reveals promising advancements along with numerous challenges and limitations. The diverse range of approaches, from image analysis to molecular profiling and risk prediction, demonstrates the versatility of AI in addressing various aspects of contact dermatitis diagnosis and patch testing in general.

The high accuracy achieved by CNN-based models in analyzing patch test images is particularly significant. With accuracies ranging from 90.1% to 99.5%, these models show great potential for automating and standardizing patch test interpretation, as some studies have shown interrater variability in diagnosing patch test reactions [4]. This could lead to more consistent diagnoses across different clinical settings, reduce dermatologists’ workload, and help expand access to patch testing. One key barrier is the need for standardized imaging protocols [8]. The variability in the quality of images, as well as the inconsistency in how and when these images are captured, introduces a significant source of error in AI models. Standardized, high-quality image capture and storage protocols are essential for ensuring that AI systems can be effectively trained and applied across different clinical settings [22].

Moreover, our review underscores the necessity for large, diverse, and representative image databases to train AI models [22,24]. Specific areas researchers should focus on include the inclusion of patients across all Fitzpatrick skin types to address potential performance gaps in darker skin tones, which are often underrepresented in dermatologic datasets [30]. The development of datasets such as the Diverse Dermatology Images (DDI) dataset underscores this need. The DDI dataset includes representation across FST I-VI for biopsy-proven correlates of benign and malignant lesions, common dermatological conditions, and ambiguous lesions [30]. Their DDI research highlighted worsened performance in the ability of certain state-of-the-art dermatology AI algorithms to accurately diagnose skin conditions in darker skin tones of FST V-VI. Their research also found that fine-tuning on diverse image sets such as DDI could overcome the gap in performance of the AI algorithms when comparing FST I-II and V-VI. Ensuring geographic and demographic diversity by collaborating with institutions in varied regions globally can help capture heterogeneity in environmental exposures, allergen profiles, and clinical practices. Initiatives such as federated learning offer a multi-institutional collaborative effort to train AI algorithms while preserving institutional data privacy through a consensus model [31]. Federated learning-trained AI models have been shown to outperform models trained on single-institutional data [32]. Additionally, datasets should aim to balance age, gender, and clinical variations in skin reactions, such as weak versus strong positive reactions, to improve model robustness [33,34]. Synthetic data offer an increasingly used solution to build larger, more robust training datasets; however, special attention is needed to ensure the inclusion of diverse synthetic input to mitigate sample selection bias [35,36]. The creation of a global patch testing image repository would not only improve AI model performance but could potentially also accelerate the discovery of new dermatological insights, enable the continuous refinement of diagnostic algorithms, and increase diagnosis assistance for complex cases, especially in lower resource settings.

The application of other techniques such as RF, GB, and LR in risk prediction and biomarker discovery is also promising. The study by Cunningham et al [27], which found GB to outperform other methods in predicting patch testing results, suggests that more complex, nonlinear approaches may be necessary to capture the intricacies of skin sensitization mechanisms. This highlights the potential of ML in discerning subtle patterns that may not be apparent through traditional statistical analyses. A more widespread and diverse dataset would not only enhance the performance of AI but also address concerns around bias, ensuring that AI-driven diagnostic tools are equitable and effective for all patients, regardless of demographic factors [30].

Despite the promising results, several limitations were identified across the reviewed studies. First, most studies had relatively small sample sizes, with 8 out of 10 studies including fewer than 250 participants, and only 2 studies including more than 1000 patients [27,29]. This limits the generalizability of findings and may lead to overfitting in ML models, as many of the studies noted [24,27]. Second, there was a lack of diversity in skin types reported across studies, with 7 of our studies not specifying the range of Fitzpatrick skin types included. This is particularly important given that skin reactions can present differently across various skin types, potentially affecting the performance of image-based AI models [37,38]. Additionally, the lack of standardization in methodology across studies makes direct comparisons challenging. Some studies used standard European baseline series allergens, while others used specific sets of allergens, making it difficult to assess and compare the robustness of the models across different allergen panels.

The ethical implications of using AI in clinical practice and industry engagement in this space also warrant attention. As AI tools become more integrated into dermatology, it is crucial to maintain transparency and interpretability in AI models [39]. For successful implementation in patch-testing diagnostics, the AI system should provide clinicians with transparent, mechanism-based explanations of its predictions, including which clinical features or biomarkers are driving its decision-making process and model confidence [40]. Efforts to increase AI literacy among health care professionals, as well as to develop user-friendly AI interfaces, will be essential in fostering the integration of these technologies into routine clinical workflows.

Furthermore, the regulatory landscape for AI in dermatology, and health care more broadly, is still evolving. While AI tools show promise, rigorous validation and regulatory approval are needed before they can be fully integrated into clinical practice [41]. Dermatologists, health care institutions, and national and international policymakers must collaborate to develop clear guidelines for the safe and effective use of AI in patch testing and other dermatological applications.

Overall, AI holds immense potential to revolutionize the diagnosis of contact dermatitis through more accurate and standardized patch testing methods. However, to realize this potential, further research is needed to address the challenges of standardization, data diversity, model transparency, and regulatory oversight. With concerted efforts, AI can serve as a powerful tool in dermatology, enhancing diagnostic capabilities, improving patient outcomes, advancing precision dermatology, and ultimately contributing to more equitable health care delivery [42].

### Conclusions

This narrative review underscores the significant potential of AI to revolutionize patch testing by enhancing diagnostic accuracy, reducing inter-provider variability, and providing a more standardized, scalable system for interpreting digital patch test results. The high accuracies achieved by CNN models in patch test image analysis are particularly noteworthy, suggesting a possible path towards more standardized and objective patch test interpretation internationally. Our analysis also highlights a need for the development and adoption of standardized protocols for capturing patch test images. Establishing these protocols is crucial for facilitating accurate diagnostics across diverse patient populations, supporting quality improvement efforts, and promoting AI-driven advancements and analyses. The creation of expansive patch testing databases and standardized protocols will enable increased application of AI systems to deliver more accurate, equitable, and scalable care in the management of ACD.

## Supplementary material

10.2196/67154 Multimedia Appendix 1 Search terms used for this review.

10.2196/67154 Multimedia Appendix 2 Image datasets.

10.2196/67154 Checklist 1 PRISMA-P checklist.

## Abbreviations:

ACD: allergic contact dermatitis
AI: artificial intelligence
AUC: area under the curve
CNN: convolutional neural network
DDI: Diverse Dermatology Image
DL: deep learning
EMBASE: Excerpta Medica Database
GB: gradient boosting
ICDRG: International Contact Dermatitis Research Group
LR: logistic regression
ML: machine learning
MOAHLFA: Male-Occupational-Atopic-Hand-Leg-Face-Age
PRISMA: Preferred Reporting Items for Systematic Reviews and Meta-Analyses
RF: random forest
