# Supplementary material for: Artificial Intelligence in Patch Testing: Comprehensive Review of Current Applications and Future Prospects in Dermatology
Source: JMIR Dermatol. 2025 Jun 2;8:e67154. doi: 10.2196/67154 (PMC12178223; doi:10.2196/67154)
Supplement: Multimedia Appendix 1 [file derma-v8-e67154-s001.pdf]

Multimedia Appendix: Search terms used for this review.

Category	Search Terms
**Machine learning**	("Machine Learning"[Mesh] OR "Artificial Intelligence"[Mesh] OR "Deep Learning"[Mesh] OR "Supervised Machine Learning"[Mesh] OR "Support Vector Machine"[Mesh] OR "Unsupervised Machine Learning"[Mesh] OR "Computer Heuristics"[Mesh] OR "Natural Language Processing"[Mesh] OR "Neural Networks, Computer"[Mesh] OR "Expert Systems"[Mesh] OR "Fuzzy Logic"[Mesh] OR "Machine Learning" OR "Artificial Intelligence" OR "Deep Learning" OR "Augmented Intelligence" OR "Large Language Models" OR "Foundation Models" OR "Neural Networks" OR "Convolutional Neural Networks" OR "Supervised Learning" OR "Unsupervised Learning" OR "Natural Language Processing" OR "Image Analysis" OR "Pattern Recognition" OR "Data Mining" OR "Decision Support Systems" OR "Machine Intelligence" OR "Cognitive Computing" OR "Automated Diagnosis" OR "Predictive Modeling" OR "Computer Vision" OR "Image Recognition" OR "Algorithmic Diagnosis" OR "Ensemble Learning" OR "Health Informatics" OR "Diagnostic Decision Support" OR "Expert Systems" OR "Computer-Aided Diagnosis" OR "reinforcement learning" OR "Generative Adversarial Network")
**Patch Test**	("Patch Tests"[Mesh] OR "patch test" OR "patch testing" OR "patch tests" OR "Patch Test Allergens" OR “Drug Patch Test” OR epicutaneous test OR Contact Sensitization Testing)
**Skin**	(skin OR epicutaneous OR cutaneous OR epidermis OR “skin barrier” OR skin inflammation OR skin sensitization)
**Contact Dermatitis**	("Dermatitis, Contact"[Mesh] OR "contact dermatitis" OR "Dermatitis, Allergic Contact"[Mesh] OR Allergic Eczematous Dermatitis OR Allergic Contact Dermatitis OR "Dermatitis, Photoallergic"[Mesh] OR Photocontact Dermatitis OR Photosensitive Dermatitis OR photoallergic OR photoallergy OR "Allergic Dermatitis" OR "Contact Allergy" OR "Contact Sensitivity" OR "Contact Hypersensitivity" OR Dermatitis Allergic Occupational OR "Dermatitis, Occupational"[Mesh] OR Industrial Dermatosis OR "occupational dermatitis" OR dermatitis)

This is a Multimedia Appendix to a full manuscript published in the Journal of Medical Internet Research (JMIR) Dermatology. For full copyright and citation information see http://dx.doi.org/10.2196/67154.
