# Supplementary material for: Artificial Intelligence in Patch Testing: Comprehensive Review of Current Applications and Future Prospects in Dermatology
Source: JMIR Dermatol. 2025 Jun 2;8:e67154. doi: 10.2196/67154 (PMC12178223; doi:10.2196/67154)
Supplement: Multimedia Appendix 2 [file derma-v8-e67154-s002.pdf]

Multimedia Appendix: Image datasets.

Author, Year	Image Modality	Image Acquisition and Pre-Processing	Image Format and Storage
Ravishankar et al., 2024 [21]	Teledermatology submissions (during COVID-19 pandemic).	Cropping into individual patch images with ImageJ, an open-source image processing software.	Portable Network Graphic (PNG).
Hall et al., 2024 [22]	Canon 5D Mark 4 digital camera, iPhone XR smartphone camera by medical photographer.	Approximately 200 x 80 pixels (Canon 5D Mark 4 digital camera) or 828 x 1792 pixels (iPhone XR smartphone camera).	Resized to 160 x 64 color pixels.
Chan et al., 2021 [26]	12-megapixel camera.	Automated image cropping into individual allergens, normalized by temperature, hue, and intensity.	Resolution normalized.
Vezakis et al., 2023 [24]	Antera 3D® camera, an advanced multi-modal imaging device.	With Antera 3D® software, multi-modal imaging (color, redness, texture, fine lines, volumes) were constructed into two- and three-dimension analyses of skin surfaces.	480 x 480 pixels, Joint Photographic Experts Group (JPEG).

This is a Multimedia Appendix to a full manuscript published in the Journal of Medical Internet Research (JMIR) Dermatology. For full copyright and citation information see http://dx.doi.org/10.2196/67154.
